# Hypothesis and Theory: Characterizing Abnormalities of Energy Metabolism Using a Cellular Platform as a Personalized Medicine Approach for Alzheimer’s Disease

**DOI:** 10.3389/fcell.2021.697578

**Published:** 2021-07-30

**Authors:** Woo-In Ryu, Bruce M. Cohen, Kai-C. Sonntag

**Affiliations:** ^1^Department of Psychiatry, McLean Hospital, Harvard Medical School, Belmont, MA, United States; ^2^Basic Neuroscience Division, McLean Hospital, Harvard Medical School, Belmont, MA, United States; ^3^Program for Neuropsychiatric Research, McLean Hospital, Harvard Medical School, Belmont, MA, United States; ^4^Psychotic Disorders Division, McLean Hospital, Harvard Medical School, Belmont, MA, United States

**Keywords:** Alzheimer’s disease, bioenergetics, metabolism, pluripotent stem cell (PSC), cellular platform, disease modeling, personalized medicine

## Abstract

Sporadic or late-onset Alzheimer’s disease (LOAD) is characterized by slowly progressive deterioration and death of CNS neurons. There are currently no substantially disease-modifying therapies. LOAD pathology is closely related to changes with age and include, among others, accumulation of toxic molecules and altered metabolic, microvascular, biochemical and inflammatory processes. In addition, there is growing evidence that cellular energy deficits play a critical role in aging and LOAD pathophysiology. However, the exact mechanisms and causal relationships are largely unknown. In our studies we tested the hypothesis that altered bioenergetic and metabolic cell functions are key elements in LOAD, using a cellular platform consisting of skin fibroblasts derived from LOAD patients and AD-unaffected control individuals and therefrom generated induced pluripotent stem cells that are differentiated to brain-like cells to study LOAD pathogenic processes in context of age, disease, genetic background, cell development, and cell type. This model has revealed that LOAD cells exhibit a multitude of bioenergetic and metabolic alterations, providing evidence for an innate inefficient cellular energy management in LOAD as a prerequisite for the development of neurodegenerative disease with age. We propose that this cellular platform could ultimately be used as a conceptual basis for a personalized medicine tool to predict altered aging and risk for development of dementia, and to test or implement customized therapeutic or disease-preventive intervention strategies.

## Introduction

Alzheimer’s disease currently affects 6.2 million Americans age 65 and older and more than 50 Million people worldwide (Alzheimers Dement, 2021). In the United States, this number is projected to triple in 2060 with an estimate of 13.8 Million AD patients posing an immense burden on the public health, economic, and social systems. Despite substantial investment in AD research, there has been no breakthrough in developing and implementing a feasible disease-modifying or curative treatment for AD (Amtul, 2016; Long and Holtzman, 2019). Altogether, there is a clear imperative to develop effective treatment or disease-preventive options, but this will likely require a better understanding of AD pathology and broader intervention strategies than the current efforts targeting end stage plaques and tangles (Long and Holtzman, 2019).

One of the main barriers to controlling AD is the still limited understanding of its pathology. For decades the “amyloid cascade hypothesis,” proposing that an accumulation of toxic amyloid beta (Aβ) plaques and twisted strands of hyperphosphorylated *tau* (tangles) in the brain, has been the dominant hypothesis of AD pathology (Hardy and Higgins, 1992; Park et al., 2009; Mawuenyega et al., 2010; Holtzman et al., 2011). This hypothesis is best supported for familial/early-onset forms of AD (EOAD), while less so for the far more common sporadic/late-onset forms (LOAD) that occur after age 65 and comprise about 95% of AD patients (Herrup, 2015; Amtul, 2016; Harrison and Owen, 2016). In LOAD, it is less likely that Aβ-plaques and *p*-tau-tangles are primary causes of the disease and more likely that they are secondary toxic products triggered by other mechanisms. Over the years, several alternative AD-causing hypotheses have been formulated with a focus on bioenergetic, metabolic, biochemical, neurovascular, and neuroinflammatory processes, suggesting that LOAD is most likely a multifactorially determined neurodegenerative disorder (Krstic and Knuesel, 2013; Herrup, 2015; Amtul, 2016).

The main risk factor for LOAD is age and a key alteration in aging is change in bioenergetics, i.e., cellular energy metabolism to produce ATP. Altered bioenergetics and cellular energy deficits are part of the normal aging process and have also been implicated in the pathophysiology of AD and other neurodegenerative diseases as well (Liang et al., 2008; Imai and Guarente, 2014; Cha et al., 2015; Beck et al., 2016; Bonkowski and Sinclair, 2016; Mastroeni et al., 2017; Sonntag et al., 2017; Sorrentino et al., 2017). However, data demonstrating causative connections with AD pathogenic processes are still sparse, partly due to a lack of model systems that recapitulate the human condition. To overcome this deficit, we have collected dermal fibroblasts and blood samples from LOAD patients and AD-unaffected control individuals and derived induced pluripotent stem cells (iPSC) to generate brain-like cells as a platform to investigate cellular processes in LOAD in context of age, disease, genetic background, cell development, and cell type. Using this *in vitro* cell model on a limited number of samples, we have recently observed that LOAD cells are consistently associated with a multitude of inherent bioenergetic and metabolic alterations (Sonntag et al., 2017; Ryu et al., 2021). Here, we provide a comprehensive representation of this platform using a larger sample number of fibroblasts and iPSC-derived neural progenitor cells (NPCs) and astrocytes to substantiate our hypothesis of an innate abnormal and inefficient cellular energy management in LOAD that likely predisposes to altered aging and the development of neurodegenerative disease. Conceptually, we propose that this cellular platform could be a diagnostic tool to identify genetic, bioenergetic, and metabolic risk factors predicting aberrant aging or dementia development, as well as a tool to identify targets and test or implement customized therapeutic or disease-preventive intervention strategies, as a personalized medicine approach for LOAD.

## Load Is a Multifactorially Determined Neurodegenerative Disorder Closely Related to Age

While EOAD is associated with genetic mutations in the amyloid precursor protein (APP) and factors involved in its processing, such as presenilins (PSEN), leading to accumulation of Aβ-plaques and *p*-tau-tangles and death in mid-life, these mechanisms are less supported as underlying LOAD (Herrup, 2015; Amtul, 2016; Harrison and Owen, 2016). In fact, there is increasing evidence that in LOAD, the accumulation of toxic Aβ and *p*-tau molecules may not be the sole or initial cause of the disease and may instead largely be the consequence of other causative factors (Krstic and Knuesel, 2013; Trushina et al., 2013; Herrup, 2015; Amtul, 2016; Harrison and Owen, 2016; de la Monte, 2017). Because aging itself is one of the major determinants of LOAD, many pathological events in LOAD are also part of the normal aging process (Mattson and Arumugam, 2018). Thus, LOAD appears to be characterized by a combination of several interacting pathological events that are closely related to changes with age and that include, among others, altered bioenergetic, metabolic, microvascular, and inflammatory processes (Wingo et al., 2012; Herrup, 2015; Amtul, 2016; Harrison and Owen, 2016; Stoeckel et al., 2016; de la Monte, 2017; Mastroeni et al., 2017; Mattson and Arumugam, 2018; Chow et al., 2019; Long and Holtzman, 2019) as well as abnormalities of cell membranes and membranous elements, including endosomes, ER, and mitochondria (Zubenko et al., 1984, 1987; Sihag et al., 1994; Wang and Zhang, 2021). Targeting and ameliorating even one of these major causative elements might reduce the occurrence or progression of LOAD. The brain requires high energy for its functions and, herein, we propose characterizing and targeting abnormalities of energy production as crucial for understanding and addressing LOAD.

## The Hypothesis: Alterations in Energy Metabolism Are Key Mechanisms Underlying Aberrant Aging and Load Dementia

**FIGURE 1:**
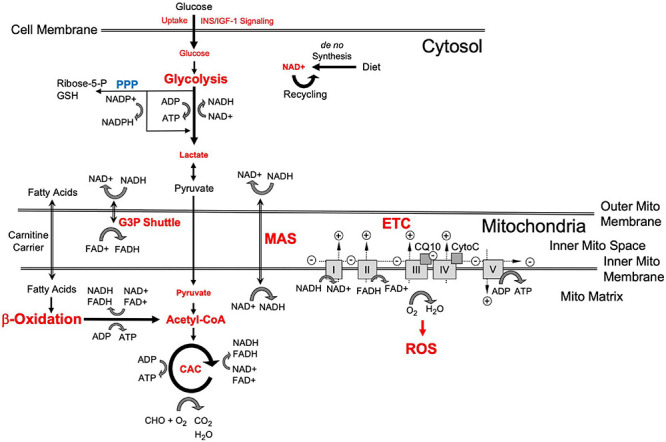
Schematic representation of cellular energy flow and substrate metabolism [adapted from Ryu et al. (2021)]. Red indicates bioenergetic processes and “branch points” where changes in late-onset Alzheimer’s disease (LOAD) cells occur as described in the text. Small script indicates LOAD-associated reductions and large script indicates increases in bioenergetic cell functions. A key metabolite is glucose whose uptake is controlled by INS/IGF-1 signaling and which is processed through glycolysis to pyruvate and may be converted to lactate. Lactate can be converted back to pyruvate, which is used in the mitochondria to produce Acetyl-CoA and oxaloacetate to fuel the citric acid cycle (CAC) that generates the reducing agents FADH_2_ and NADH. NADH is the reduced form of NAD^+^ that is newly synthesized or recycled and serves as a proton and electron transfer molecule. The protons and electrons carried by NADH and FADH_2_ are used to generate a proton gradient in the electron transfer chain (ETC), which includes complexes I–V, coenzyme Q, and cytochrome C, and leads to the production of ATP by OxPhos that produces H_2_O and reactive oxygen species (ROS). Because glycolytically produced cytosolic NADH cannot pass the outer mitochondrial membrane, protons and electrons are transported through the glycerol-3-phosphate (G3P) shuttle and malate aspartate shuttle (MAS) which generate FADH_2_ in the intermembrane space and NADH in the mitochondrial matrix, respectively. Fatty acids are transported to the mitochondria through carnitine carriers and their metabolism in β-oxidation is an alternative pathway to produce FADH_2_, NADH, ATP, and Acetyl-CoA. The pentose phosphate pathway (PPP) produces ribose-5- and xylulose-5-phosphate leading to the reduction of NAPD^+^ to NAPDH that is used for fatty acid biosynthesis and regeneration of reduced glutathione (GSH) to neutralize ROS.

Bioenergetics is “the biology of energy transformations and energy exchanges within and between living things and their environments^1^.” This energy flow consists of a multitude of different cellular systems and processes that include glycolysis, respiration and other forms of metabolism, to produce and utilize energy (Figure 1). A major energy substrate is glucose, which is metabolized via cytosolic aerobic glycolysis to pyruvate or under low oxygen conditions via anaerobic glycolysis to lactate. Pyruvate, directly obtained from glycolysis or converted from lactate, is processed to Co-enzyme A (CoA) to drive the tricarboxylic or citric acid cycle (TCA and CAC) and support oxidative phosphorylation (OxPhos). In the mitochondria, OxPhos is coupled to CAC and can metabolize substrates from different sources, including carbohydrates (i.e., pyruvate or lactate converted from glucose), ketones and fatty acids that are processed through β-oxidation to produce CoA, glutamine, and other substrates. Both glycolysis and mitochondrial respiration produce the reduced form of nicotinamide adenine dinucleotide (NADH) from NAD^+^, an essential oxidizing agent involved in redox reactions and electron transfer. The mitochondrial processes entirely depend on oxygen and are highly efficient, producing about 34–36 mol of ATP from one mol glucose with CO_2_ and H_2_O as waste products. In contrast, both human forms of glycolysis generate ATP at low efficiency (about two mol per mol of glucose), though under certain conditions aerobic glycolysis can produce ATP faster and, thus, be more efficient than OxPhos. A third mechanism to metabolize glucose is through the pentose phosphate pathway (PPP), in which glucose-6-phosphate is converted to ribose-5- and xylulose-5-phosphate leading to the reduction of NAPD^+^ to NAPDH that is used for fatty acid biosynthesis and regeneration of reduced glutathione which neutralizes reactive oxygen species (ROS) produced by OxPhos. In the brain, astrocytes can also store glucose in the form of glycogen, which is a substrate for glycolysis when needed.

As stated in our previous publication (Ryu et al., 2021) and elsewhere, eukaryotic cells use about 10% glycolysis and 90% OxPhos for energy production, with proportions depending on cell type and metabolic state (Mookerjee et al., 2017). In particular, growth or the need for rapid production of energy leads to increased production of ATP by glycolysis. The brain consumes about 20% of total body oxygen and utilizes 25% of total body glucose, extracting approximately 50% of oxygen and 10% of glucose from the arterial blood. Aerobic glycolysis is the major mechanism of energy production in the brain, with astrocytes consuming about 85% of the glucose utilized brain-wide to generate pyruvate and release lactate for use by neurons (Bordone et al., 2019; Dienel, 2019). These high energy needs make the brain especially susceptible to energy production and flow disruptions, which could be important considerations in brain aging and neurodegeneration. It is well documented that mitochondrial functions decrease with age, and many brain disorders, including AD, show evidence of abnormal energy metabolism (Swerdlow and Khan, 2004; Kapogiannis and Mattson, 2011; Jove et al., 2014; Sun et al., 2016; Mattson and Arumugam, 2018; Butterfield and Halliwell, 2019; Hou et al., 2019; Huo et al., 2020). In fact, bioenergetic deficits may be a key causative event in the pathogenesis of LOAD, leading to neuronal vulnerability and diminishing the neuroprotective capacities of the brain, thus, enhancing and accelerating neurotoxic effects through oxidative metabolites and neuronal excitability, and susceptibility to other general or disease-specific stressors. Specifically, age-related and disease-specific mitochondrial damage leads to reduced efficiency of OxPhos, with neurons increasing OxPhos activity to compensate, which through altered redox reactions may abnormally upregulate glycolysis. This could be a consequence of numerous interacting factors, including mitochondrial dysfunction, in part through age-associated mitochondrial DNA deletions (Popadin et al., 2014; Taylor et al., 2014), and also through AD risk factors, such as Apoε4, which has been associated with altered cellular membrane composition and dysfunctional bioenergetics (Wu et al., 2018; Area-Gomez et al., 2020) by damaging the oxidative/mitochondrial system, in particular (Kharrazi et al., 2008; Maruszak et al., 2011; Chico et al., 2013). Mitochondrial oxidative stress is an early event in AD (Nunomura et al., 2001) and produces toxic ROS that damage DNA and other cell elements. Oxidative stress has also been implicated in damage to the brain’s vascular endothelium in early stages of AD, leading to microvascular disease and blood brain barrier dysfunction as significant additional contributors to LOAD pathology (Iadecola, 2004; Di Marco et al., 2015). In addition, astrocytes may increase metabolic rates through upregulation of glycolysis to support energy demands in neurons, resulting in an increased bioenergetic dependence of neurons on astrocytes. The increase in metabolic rates in astrocytes leads to molecular changes in these cells and may induce astrogliosis, further contributing to AD pathology (Jackson et al., 2014; Batarseh et al., 2016; Osborn et al., 2016; Sollvander et al., 2016). The concept of astrocyte/neuron-interdependent bioenergetics has been summarized in several reviews (Belanger et al., 2011; Fu and Jhamandas, 2014). The relative prominence of OxPhos has been denoted by Demetrius et al. as the “inverse Warburg effect” hypothesis based on terminology used in cancer biology (Demetrius and Simon, 2012; Demetrius and Driver, 2013; Demetrius et al., 2014). In cancer, the predominant use of glycolysis versus OxPhos is called the “Warburg effect” referring to German chemist and physiologist Otto Heinrich Warburg (1956) who discovered this metabolic switch in the 1920s, for which he received the Nobel Prize in 1931. The “inverse Warburg effect” hypothesis postulates an age-associated increase of OxPhos in neurons, probably to increase output from damaged mitochondria, and the compensatory increase of glycolysis in astrocytes to supply neurons with lactate as energy source. The bioenergetic interdependence between astrocytes and neurons is also closely linked to the glutamate/glutamine shuttle, in which glutamate is produced in neurons from glutamine that, in turn, is synthesized in astrocytes from glutamate which they take up through the glutamate transporter 1 (EEAT1) (Rose et al., 2013). Glutamate transport in astrocytes protects against the toxic effects of glutamate at the most common and greatest utilizer of energy among brain synapses, glutamatergic synapses. The high energy-consuming process of glutamate recycling is closely linked to and dependent on glycolysis. Glutamate levels are tightly regulated in the brain and disturbances of glutamate homeostasis that include excessive (glutamate excitotoxicity) or diminished stimulation of glutamatergic signalling on neurons has long been associated with neuronal dysfunction and death in AD (Wang and Reddy, 2017).

Among the consequences associated with bioenergetic disturbances in brain cells are an increase in oxidative stress and ROS produced by dysfunctional mitochondria, further damage to mitochondria, their proteins, their membranes and DNA, fuel shortage, and other downstream effects, such as protein instabilities, diminished catalytic activities, changes in protein conformation, misfolded structures and aggregation, and dysfunctional energy kinetics, all together leading to neuronal and synaptic damage and loss (Demetrius et al., 2014; Mattson and Arumugam, 2018). While normal aging is characterized by a “quasi-stable equilibrium state” between intact and dysfunctional neurons, pathological aging is induced by its disruption as a consequence of insult and loss of protection. All these processes are influenced and worsened by various metabolic and environmental factors, such as toxins, poor diet, diabetes, hypertension, and more (Scarmeas et al., 2006; Bangen et al., 2015; Lecci et al., 2015).

Many AD-pathogenic changes occur concomitantly in the aging brain, and they iteratively interact, rather than being strictly causative of one another. Aging is a dynamic process and dependent on each individual’s genetic blueprint, biological condition, environment, and lifestyle, but a central driving force in aging is how energy is produced and utilized (Mattson and Arumugam, 2018). In fact, the need to meet energy demands influences the state and activity of all other cellular functions, and because of its many fold higher energy requirements than the rest of the body, this need is more pronounced in the brain (Belanger et al., 2011; Magistretti and Allaman, 2015; Mattson and Arumugam, 2018). In LOAD, disturbances in bioenergetics and metabolic deficiencies may progressively occur as a function of normal aging and weaken the cellular responses to stress. In support of this notion, there is increasing evidence that changes in eating patterns and lifestyle and associated increases in calory intact leading to shifts in metabolism and rises in obesity and metabolic disorders, such as diabetes, have detrimental impact on the organism-wide capacity to prevent and repair cellular and molecular damage (Mattson et al., 2014; Stoeckel et al., 2016; de la Monte, 2017; Neth and Craft, 2017; Chow et al., 2019). Although these disturbances can often be compensated, at least for a while, to maintain homeostasis, they also profoundly impair the cellular stress responses leading to vulnerability toward other non-specific (e.g., neuroinflammation) and “AD-specific” (Aβ peptides, *p*-tau) toxicity, with the caveat that the term “AD-specific” may be misleading, as accumulation of Aβ plaques and *p*-tau tangles appear to be part of the normal aging process and are not necessarily tightly associated with dementia (Braak and Braak, 1997; Price et al., 2009; Rodrigue et al., 2012). Eventually, there is a tipping point when the system goes out of balance and pathology accelerates, resulting in synaptic and neuronal damage and loss.

## A Cellular Platform for Load

**FIGURE 2:**
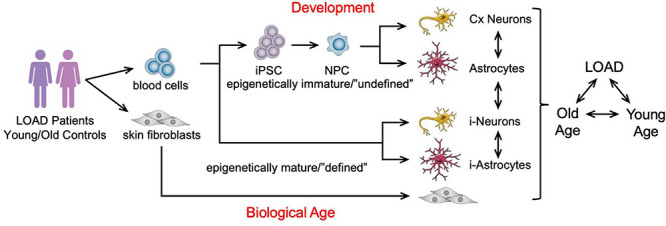
Schematic representation of an *in vitro* cellular platform for studying LOAD. LOAD patients’- and control individuals’-derived fibroblasts or blood cells are used to generate induced neurons (*i*-Neurons) or astrocytes (*i*-Astrocytes), and iPSCs for the production of NPCs, neurons, and astrocytes to study LOAD in context of brain cell development, cell types, function, and age. The cell samples analyzed in our studies are summarized in Supplementary Table 1.

There is currently no model system for LOAD that recapitulates the complexity of the disease pathology in context of the human brain and age. Since LOAD is tightly associated with one’s individual genetic precondition, course of aging, and lifestyle, it is difficult to develop adequate and comprehensive model systems. Current models are designed to analyze certain aspects of the disease, such as the impact of AD genetic mutations and target molecules in cell culture and animals or the status of the human brain in imaging or postmortem brain analyses. Although studies in such systems have revealed much data to better understand AD pathogenesis, there is still a need for experimental platforms that can integrate multiple factors linked to the human condition as a tool to address complex hypotheses. For example, it is still difficult to analyze *bona fide* human brain cells and their interaction both *in vitro* and *in vivo*. Intriguingly, the groundbreaking work by Takahashi and Yamanaka (2006) to reprogram terminally differentiated somatic tissue to iPSCs has led to the development of new technologies and study designs based on the generation of not only disease-specific but also customized patient-specific stem cells that can be differentiated into any cell type of the body for investigative or therapeutic purposes (Karagiannis and Eto, 2016; Mertens et al., 2016; Takahashi and Yamanaka, 2016; Poon et al., 2017; Sonntag et al., 2018). Experiments on EOAD and LOAD patients’ iPSCs-derived brain cells have replicated several known aspects of AD pathogenesis previously discovered in other model systems (Israel et al., 2012; Kondo et al., 2013; Duan et al., 2014; Lee et al., 2014, 2016; Mahairaki et al., 2014; Muratore et al., 2014; Sproul et al., 2014; Hossini et al., 2015; Balez et al., 2016; Raja et al., 2016; Woodruff et al., 2016; Armijo et al., 2017; Jones et al., 2017; Martin-Maestro et al., 2017; Ochalek et al., 2017; Oksanen et al., 2017; Yang et al., 2017; Zhao et al., 2017; Kwart et al., 2019; Meyer et al., 2019). In addition, fibroblasts or other mature cells can be directly converted to induced neurons (iN) or astrocytes (*i*-astrocytes) adding another layer to cell culture paradigms by bypassing cell development and preserving the biological age of the donor cells (Vierbuchen et al., 2010; Meyer et al., 2014; Mertens et al., 2015). However, because direct conversion retains age and lifestyle-related epigenetic changes, this approach cannot be used to detect and study innate factors underlying LOAD. To investigate innate abnormalities of bioenergetics and metabolism in LOAD, we have extended the iPSC technology to generate LOAD patients’–and control individuals’-derived neural cell populations of high phenotype integrity with concomitant studies on fibroblast cell lines, including from the same individual, to integrate cell type, development, individual genetic background, disease, and age in our studies (Figure 2 and Supplementary Table 1; Yoshimizu et al., 2015; Sonntag et al., 2017; McPhie et al., 2018; Ryu et al., 2021).

## Identification of Bioenergetic and Metabolic Alterations in Load Cells as a Function of Cell Type, Development, Genetic Background, Age, and Disease

A key element in bioenergetic processes is the redox agent NAD^+^ which is in a constant state of uptake, synthesis, degradation, and recycling [reviewed in Canto et al. (2015) and Verdin (2015)]. NAD^+^ levels are maintained either by *de novo* synthesis from tryptophan or via the salvage pathway that utilizes dietary NAD precursors, such as nicotinamide (NAM), nicotinamide mononucleotide (NMN), nicotinic acid (NA), and nicotinamide riboside (NR). The reduced form of NAD^+^, NADH, plays a critical role as an energy-transfer intermediate and is an important substrate of the mitochondrial electron transport chain (ETC), where it is oxidized to NAD^+^ donating electrons and protons during mitochondrial respiration. In normally functioning biosynthetic processes, the redox ratio (RR = NAD^+^/NADH) in the brain is balanced and the ETC is sufficiently powered for ATP production. NAD^+^ is an essential component in all bioenergetics processes and is a co-enzyme for key factors in cellular functions that have been implicated in the aging process (Canto et al., 2015; Verdin, 2015). Corrupted NAD^+^ recycling impairs glycolysis and OxPhos. In turn, enhanced NAD^+^ metabolism has been shown to protect neurons from degeneration, including in AD (Liu et al., 2008; Verdin, 2015; Lautrup et al., 2019; Braidy and Liu, 2020).

**FIGURE 3:**
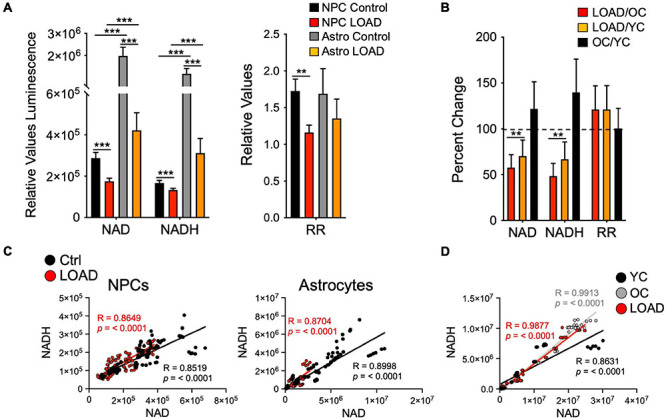
NAD^+^ and NADH production in iPSC-derived NPCs and astrocytes, and fibroblasts. **(A,B)** Relative NAD^+^ and NADH levels measured as luminescence and RR of LOAD (*n* = 9, red bars) or Control (*n* = 9, black bars) NPCs, and LOAD (*n* = 9, orange bars) or Control (*n* = 9, gray bars) astrocytes **(A)**, or as percent change LOAD/OC, LOAD/YC, and OC/YC from *n* = 16 YC, *n* = 14 OC, and *n* = 11 LOAD fibroblasts **(B)**. The average ages for fibroblasts are YC 26.5 ± 7.1, OC 67 ± 8.8, and LOAD 69 ± 9.7. **(C,D)** Pearson’s correlation coefficients for NAD^+^ and NADH. Linear correlations for Control and LOAD NPCs and astrocytes **(C)**, and fibroblasts **(D)** for NAD^+^ and NADH, plotted as relative values luminescence. R and *p* values are indicated. Data are means +/– SEM from 2 to 4 repeat experiments. *p < 0.1; **p < 0.05; ***p < 0.01 using one-way ANOVA.

It has been observed that NAD^+^ levels, themselves, and factors in its synthesis and/or recycling, such as nicotinamide mononucleotide adenylyl transferases (NMNAT) that produce the NAD precursor nicotinic acid adenine dinucleotide (NADD), diminish with age and in neurodegenerative diseases, including LOAD (Verdin, 2015; Zhu et al., 2015; Ali et al., 2016; Lautrup et al., 2019). In conjunction with decreased activity of NAD^+^ signaling proteins, this decline is believed to be one of the major reasons why organisms age (Rajman et al., 2018). In our cell platform, we consistently found a reduction of NAD^+^ levels in LOAD iPSC-derived NPCs and astrocytes and fibroblasts when compared to control samples (Figures 3A,B and Supplementary Figure 1; Sonntag et al., 2017; Ryu et al., 2021). Concomitantly, NADH levels are also reduced in LOAD samples and the RR are lower in LOAD NPCs and astrocytes but not in fibroblasts. Importantly, regression analyses revealed overall positive correlations between NAD^+^ and NADH demonstrating that despite lower total NAD^+^ levels, the overall reducing power is not diminished in LOAD cells (Figures 3C,D). As for cell development and cell phenotype determination, astrocytes have higher NAD^+^/NADH levels than NPCs, consistent with elevated metabolic rates during development (see below). Accumulating evidence has demonstrated that a steady decline in NAD^+^ levels over time is a natural part of life for all species (Verdin, 2015; Zhu et al., 2015; Rajman et al., 2018). In humans, this decline occurs rapidly after age 45 (Imai and Guarente, 2014). The consequences of a decline in NAD^+^ levels could be compromised mitochondrial energy production and impairment of the clearance of damaged mitochondria (Papa and Germain, 2017), along with a diminished capacity of the brain’s response to oxidative stress (Kharrazi et al., 2008; Maruszak et al., 2011; Chico et al., 2013; Das et al., 2018; Liguori et al., 2018; Stapper and Jahn, 2018). States of intensive compensatory energy metabolism would lead to the production of ROS, which increase in an age-dependent fashion within the mitochondria (Kuka et al., 2013) with subsequent toxic effects damaging proteins, lipids, and DNA, eventually resulting in neuronal death. It should be noted that consistent with previous observations (Chapman et al., 1983) the fibroblasts from old control (OC) subjects in our study had higher NAD^+^ levels than seen in cells from young control (YC) subjects (Figure 3B). Regression analysis showed strong positive correlations of NAD^+^ and NADH with age in YC (ages 21–37) but not in OC (ages 55–82) or YC+OC fibroblasts, and there were negative correlations in LOAD cells (ages 56–81) (Supplementary Figure 2). Interestingly, RR correlated positively with age in LOAD fibroblast, while there were trends of negative correlations in YC and OC. Despite the consensus in the field that age in general is associated with a systemic decline of NAD^+^, the data on the fibroblasts indicate that this may not equally apply to all cellular phenotypes, such as the skin fibroblast population analyzed here, in which a combination of both age and a LOAD background appears to be the driving factor for a decline in NAD^+^ and NADH.

To characterize the bioenergetic status of cells in our platform, we used Seahorse Mito Stress Tests, which determine the oxygen consumption rate (OCR, pmol/min) and the extracellular acidification rate (ECAR, mpH/min) after injection of specific pharmacologic stressors that target the ETC and ATP production, including oligomycin, which inhibits complex V (ATP synthase), decreasing OCR and ATP production; carbonyl cyanite-4 (trifluoromethoxy) phenylhydrazone (FCCP), which disrupts the mitochondrial membrane potential and collapses the proton gradient at the ETC leading to maximal respiration (O_2_ consumption by complex IV); and rotenone/antimycin A, which inhibit complex I and III resulting in the shutdown of mitochondrial respiration. The tests reveal the cells’ oxidative parameters, including basal and maximal respiration, their spare respiratory capacity (maximal respiration minus basal respiration), proton leak, non-mitochondrial respiration, and the coupling effect, which determines ATP production relative to basal respiration. In addition, the ECAR from which the proton efflux rate (PER, pmol H^+^/min) can be calculated is an indirect measure of lactate production and, thus, the cells’ glycolytic capacity. Finally, the cellular respiratory or glycolytic response to the pharmacological-induced mitochondrial stress can be determined as OCR or ECAR metabolic potentials, respectively.

**FIGURE 4:**
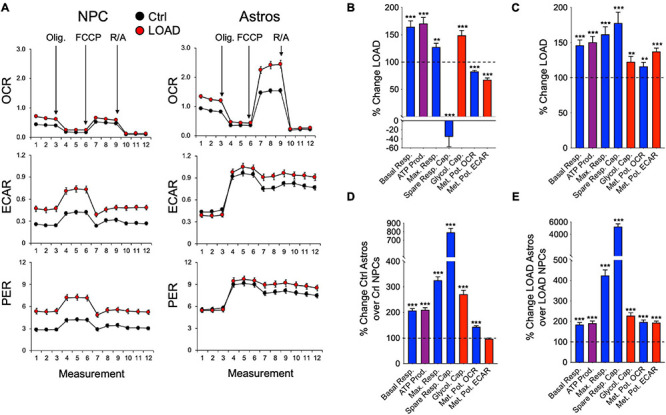
Bioenergetic profiles of iPSC-derived NPCs and astrocytes. **(A)** Profiles of Seahorse XFp Mito Stress Test data for OCR (pmol/min), ECAR (mpH/min), and PER (pmol H^+^/min) in LOAD (*n* = 9) and Control NPCs (*n* = 9) and LOAD (*n* = 9) and Control astrocytes (*n* = 9). LOAD samples are in red and Control samples in black. Arrows indicate injections of oligomycin (Olig.), carbonyl cyanite-4 (trifluoromethoxy) phenylhydrazone (FCCP), and rotenone/antimycin A (R/A). **(B–E)** Calculated values of OCR (blue) and ECAR (red) parameters and results from Cell Energy Phenotype Test Report Generator for NPCs **(B)** and astrocytes **(C)** plotted as percent change LOAD over Control, and as change Ctrl astrocytes over Control NPCs **(D)** and LOAD astrocytes over LOAD NPCs **(E)**. Data are means +/- SEM from 2 to 4 repeat experiments. *p < 0.1; **p < 0.05; ***p < 0.01 using one-way ANOVA.

**FIGURE 5:**
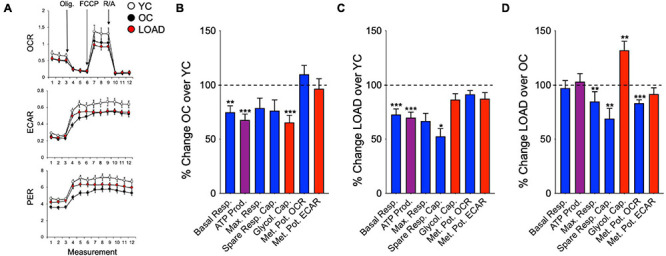
Bioenergetic profiles of fibroblasts. **(A)** Profiles of Seahorse XFp Mito Stress Test in YC (*n* = 16, average age 37.2 ± 10.7), OC (*n* = 10, average age 65.3 ± 8.2), and LOAD (*n* = 10, average age 71 ± 9.5) fibroblast lines. YC are marked in white, OC in black, and LOAD samples in red lines. **(B–D)** Calculated values of OCR (blue) and ECAR (red) parameters and results from Cell Energy Phenotype Test Report Generator plotted as percent change OC/YC **(B)**, LOAD/YC **(C)**, and LOAD/OC **(D)**. Data are means +/– SEM from 2 to 3 repeat experiments. *p < 0.1; **p < 0.05; ***p < 0.01 using one-way ANOVA.

Overall, LOAD iPSC-derived NPCs and astrocytes have heightened respiratory and glycolytic activities with increased basal and maximal respiration (Figures 4A–C). Furthermore, increased ATP production indicative of higher energy output was observed in LOAD cells. Differences are seen in spare respiratory capacity, which is diminished in LOAD NPCs but increased in LOAD astrocytes, suggesting reduced respiratory plasticity in LOAD NPCs. In addition, LOAD NPCs and astrocytes have increased glycolysis, while the respiratory and glycolytic responses to mitochondrial stress are decreased in LOAD NPCs and increased in LOAD astrocytes, attesting to a higher bioenergetic plasticity of LOAD astrocytes. In cell development, Control as well as LOAD astrocytes have similar bioenergetic profiles and show increases in respiratory and glycolytic activities when compared to NPCs, demonstrating that metabolic rates increase during cell differentiation (Figures 4D,E). However, only LOAD astrocytes exhibit elevated glycolytic plasticity in response to mitochondrial stress. In addition to iPSC-derived cells, the analysis of fibroblast lines reveals information on age-associated bioenergetic changes and whether alterations are body-wide (Sonntag et al., 2017). In age, i.e., comparing OC or LOAD with YC, there was a reduction in respiratory parameters, ATP production, and glycolysis but little change in the metabolic potentials (Figures 5A–C). However, comparing LOAD with OC, there was an increase in glycolytic capacity, while the respiratory capacity (maximal and spare capacity, and OCR metabolic potential) was decreased (Figure 5D). Overall, these data show that LOAD is associated with heightened respiratory and glycolytic activities in immature and differentiated neural cell populations without affecting cell-specific bioenergetic switches during cell development, and that increased glycolysis is also a feature of LOAD fibroblasts, while decreased glycolysis is a factor associated with age, itself.

In addition to analyzing the cells’ oxidative and glycolytic status in resting conditions, we determined their bioenergetic metabolic capacity using the Biolog MitoPlate S1 platform. These assays assess mitochondrial function by measuring the rates of electron flow into and through the ETC from 31 metabolic substrates that follow different pathways and transporters to enter the mitochondria and are processed by different dehydrogenases to produce NADH or FADH_2_. Electron transfer is then monitored by a tetrazolium redox dye which acts as a terminal electron acceptor provided by cytochrome c.

**FIGURE 6:**
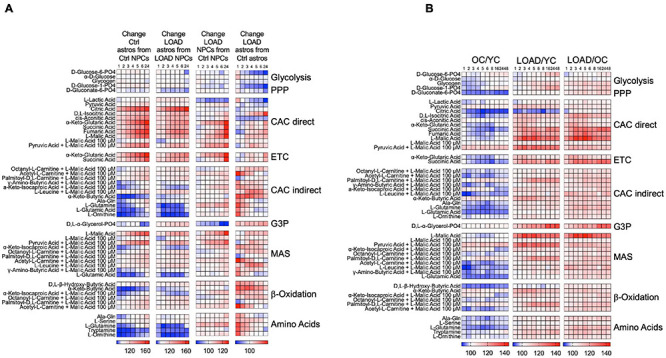
Processing of bioenergetic substrates in iPSC-derived NPCs and astrocytes, and fibroblasts. **(A,B)** Data from Biolog MitoPlate S-1 assays on LOAD (*n* = 9) and Control (*n* = 9) NPCs and astrocytes **(A)**, and YC (*n* = 7, average age 30.1 ± 8.6), OC (*n* = 7, average age 68.4 ± 10.8), and LOAD (*n* = 9, average age 70.9 ± 7.4) fibroblasts **(B)**. Processing of 31 substrates were kinetically measured at 1, 2, 3, 4, 5, 6, 8, 16, 24, and 48 h, and data plotted as heat maps showing percent changes in astrocytes from NPCs reflecting changes in cell development, LOAD from Control cells reflecting disease-associated alterations, and OC from YC reflecting age. The data are from duplicate experiments and clustered according to bioenergetic functions as explained in the text.

When we kinetically measured the processing of substrates over 24 h (NPCs and astrocytes) or 48 h (fibroblasts), we found differences in the cells’ capacity to activate bioenergetic pathways in context of cell type, development, LOAD, and age (Figure 6). In cell development, astrocytes upregulate substrate metabolism related to CAC and ETC, the malate-aspartate shuttle (MAS), β-oxidation, and to some extend glycolysis, but reduce metabolism of amino acids, α-keto-butyric and α-keto-isocaproic acid that are involved in CAC and β-oxidation (Figure 6A). Overall, there are no striking differences between developing LOAD and Control astrocytes, except for *D*,*L*-α-glycerol-phosphate [glycerol-3-phosphate (G3P)], a substrate in lipid synthesis and the glycerophosphate shuttle to transport reducing equivalents from cytosolic NADH to ETC (Mracek et al., 2013), whose metabolism is decreased in Control but increased in LOAD astrocytes. The enzyme that processes G3P is glycerol-3-phosphate dehydrogenase (GPDH), and it has been reported that mammalian astrocytes express GPDH during development and postnatally (Rust et al., 1991; Nguyen et al., 2003; McKenna et al., 2006). Together, these data demonstrate a bioenergetic and metabolic shift of developing astrocytes toward increased mitochondrial respiration (CAC, ETC, MAS, and β-oxidation activity), a reduction in amino acid metabolism, and in LOAD cells a switch to G3P shuttle activity. With regards to LOAD, in both LOAD NPCs and astrocytes there were reductions of glycolytic pathway activation and lactate processing and an activation of ETC, MAS, β-oxidation, and amino acid processing. These features are more pronounced in LOAD astrocytes, which also have reduced CAC activity. In addition, there is a striking difference in G3P shuttle activity, which is reduced in LOAD NPCs but increased in LOAD astrocytes. Since the G3P shuttle is activated by glucose metabolism and lactate [reviewed in McKenna et al. (2006)], its activation in LOAD astrocytes could be a compensatory effect to maintain sufficient mitochondrial transport of reducing agents in response to diminished glycolysis and CAC activation. The analysis of fibroblasts also showed LOAD-associated and additional age-related metabolic changes (Figure 6B). While OC had an overall reduction of substrate metabolism in both glycolysis and all aspects of the respiratory chain when compared to YC, LOAD cells exhibited an overall upregulation of these functions in comparison with both YC and OC.

Altogether, the Biolog data revealed that LOAD cells have alterations in bioenergetic functions with regards to the production or transfer of reducing agents into the mitochondria and at the interphase of glycolysis and the mitochondrial respiratory chain. In particular, it appears that a LOAD-associated phenotype in neural cells is characterized by diminished activation of glycolysis and CAC in association with reduced lactate to pyruvate conversion or metabolism causing compensatory shifts toward energy production through β-oxidation and amino acid processing. In addition, the general increase in metabolic functions in LOAD appears to be LOAD- and not age-specific, as they are also seen in LOAD fibroblasts but not in old fibroblasts from LOAD-unaffected subjects.

**FIGURE 7:**
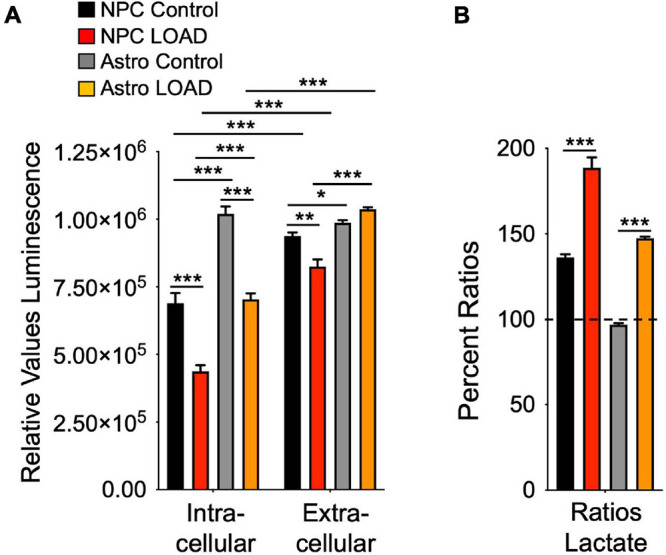
Lactate production in iPSC-derived NPCs and astrocytes. Intracellular and extracellular (media) lactate levels were measured on LOAD (*n* = 9) and Control (*n* = 9) NPCs and astrocytes sand plotted as relative values luminescence **(A)**, or as percent ratios of extracellular to intracellular lactate **(B)**. Data are means +/– SEM from duplicate experiments. *p < 0.1; **p < 0.05; ***p < 0.01 using one-way ANOVA.

The Biolog experiments indicated that despite the increased glycolytic rates in LOAD NPCs and astrocytes, their glycolytic substrate metabolism and lactate conversion was reduced. We therefore measured lactate levels in these cells as an additional indicator of glycolysis (Figure 7). Overall, astrocytes have higher lactate levels than NPCs, and there were diminished intracellular lactate levels in both LOAD NPCs and astrocytes (Figure 7A). Extracellular lactate levels were higher than intracellular levels in all cells, except for Control astrocytes, indicating lactate release, and the ratios of extracellular versus intracellular levels were higher in LOAD NPCs and astrocytes than in control cells (Figure 7B).

## Discussion

**FIGURE 8:**
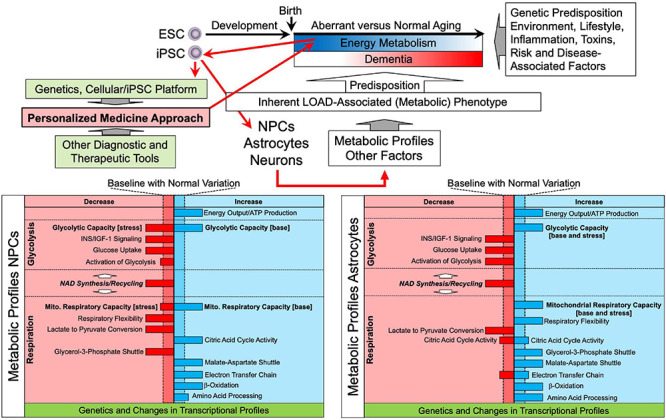
Schematic representation of LOAD-associated metabolic profiles and a personalized medicine approach to predict and prevent altered aging and dementia. Aging is characterized by alterations and reductions of energy metabolism along with the effects of multiple other factors associated with genetic predisposition, lifestyle, environment, among others, and there are numerous risk and disease-specific factors and events. The concomitant interaction of these factors determines the aging process, i.e., normal aging without the development of major dementia, or aberrant aging that may lead to dementia, including LOAD. It is still unclear when the aging process becomes pathological and triggers disease development, but it may start occurring early in life. A personalized medicine approach using the iPSC platform in combination with genetic analysis could be applied early in life, e.g., shortly after birth, to determine individual metabolic profiles and risk factors of disease. These profiles detect and identify aberrant metabolic and other processes and factors that deviate from baseline and can be used to project one’s individual aging process and risk for neurodegenerative disease, and dementia development leading to preventive or therapeutic measures, such as lifestyle recommendations or testing personalized therapeutic interventions in the cell platform. The platform can also be combined with other diagnostic or therapeutic tools to monitor or interfere in aging or disease manifestation. ESC, embryonic stem cell.

Our investigations, so far, have established that LOAD is associated with several specific and consistently observed deficits in efficiently producing energy due to impairments in numerous key components of bioenergetic substrate uptake or production along with enhanced alternative pathways for energy production and transport (Figures 1, 8). This is consistent with other evidence that risk for LOAD is associated with numerous interacting factors each contributing to outcome in some degree. In this regard, type 2 diabetes and hyperlipidemia are metabolic comorbidities in three of the 16 subjects in our LOAD cohort but in only one of the 31 subjects in the control group. As to specific abnormalities, our data confirmed and extended previous observations that INS/IGF-1 signaling and glucose uptake are compromised in LOAD (Stoeckel et al., 2016; de la Monte, 2017; Neth and Craft, 2017) by showing that this deficit already occurs in early brain development and may be a function of lower glucose transporter (GLUT1), and INS receptor densities (Sonntag et al., 2017; Ryu et al., 2021). Concomitantly, LOAD cells exhibit increased glycolysis at baseline, perhaps as consequence of reduced glucose uptake, and seem to be operating at their maximal glycolytic capacity, as they do not efficiently activate glycolysis further when exposed to glycolytic substrates. Despite heightened glycolysis, LOAD cells have diminished intracellular lactate but also seem to release lactate at a higher rate than control cells, which could be an indication of LOAD-associated differences in lactate homeostasis. Similar to glycolysis, mitochondrial respiration is increased in LOAD under baseline conditions, which appears to be a LOAD-specific effect, as OxPhos is decreased in old age cells. In development, both LOAD and Control NPCs have similar bioenergetics changes when differentiating to astrocytes. However, LOAD astrocytes exhibit increased glycolytic plasticity in response to mitochondrial stress which is indicative of a Warburg-type effect and could be an adaptive mechanism to compensate for the reduction of respiratory flexibility in LOAD NPCs during cell development. The heightened level of respiration in LOAD cells appears to be associated with modifications in the respiratory pathways, i.e., deficits in activating CAC, ETC, and in NPCs the G3P shuttle, while upregulating MAS and β-oxidation. These switches seem to be LOAD-specific, as they are less obvious in cells from subjects who are old but do not have LOAD. The bioenergetic alterations in LOAD may in part be driven by an apparent deficit in converting lactate to pyruvate and a stark reduction in total NAD^+^ levels, which would disconnect glycolysis from respiration and provide inadequate levels of redox agents in LOAD cells, respectively. In addition, as previously published, there are multiple differentially expressed genes of factors related to the observed bioenergetic alterations in LOAD cells, suggesting an innate genetic component driving these processes in LOAD (Ryu et al., 2021). Overall, it appears that LOAD cells “work harder” to produce and maintain energy balance and that these changes are inherent, partly body-wide, and already occur in early development. LOAD may, thus, be a consequence of lifelong altered and inefficient energy management leading to progressive homeostatic imbalances and associated downstream effects including the inability to sufficiently compensate for neurotoxic insults, all contributing to an aberrant aging process resulting in neural dysfunction and degeneration at the cellular level, and dementia at the cognitive level (Vlassenko et al., 2010; Mattson and Arumugam, 2018; Baik et al., 2019; Hou et al., 2019; Lautrup et al., 2019; Pacholko et al., 2019; Ryu et al., 2021). Among the consequences of these abnormalities are alterations in many cellular processes, including protein metabolism, which may lead to abnormal products, such as the plaques and tangles seen in degenerating brain in most cases of LOAD.

The notion that LOAD is caused by multifactorial events which are tightly linked to the aging process requires the implementation of integrative approaches to develop treatment options, which include early diagnosis, i.e., better understanding risk factors and the use of biomarkers before clinical symptoms occur; preventive strategies, i.e., intervention before disease onset to halt progression or delay onset; and multitarget regimens, i.e., appreciating the complexity of LOAD pathology and developing holistic therapeutic or preventive approaches. Further development and application of the techniques and findings presented here could provide a conceptual basis for a novel personalized medicine approach to detect and address altered aging and LOAD. Based on our findings that there are seemingly specific inherent LOAD-associated bioenergetic and metabolic alterations in cell functions, one could apply the iPSC platform early in life to generate individual genetic and metabolic profiles as a diagnostic tool to predict one’s aging process and risk for dementia later in life (Figure 8). These profiles could be supplemented with other age- or pathogenic parameters and factors to refine and narrow down diagnostic assessments. Findings might then be used to implement preventive or therapeutic measures, such as lifestyle recommendations and adjustments, currently, or testing personalized therapeutic interventions in the cellular platform, in the future. In addition, the platform can be used further to study the origins of LOAD pathology and identifying novel therapeutic targets, and it can be combined with other diagnostic or therapeutic tools, such as imaging technology, blood tests, drug screens, etc., to monitor and guide alterations in aging or disease manifestation. And in time, with comparisons to the characteristics of cells from patients with other neurodegenerative disorders or forms of dementia, the platform could be used to identify risks and suggest interventions for various specific life-long outcomes. Altogether, an iPSC personalized medicine approach, as described herein, could become a major advance in understanding the causes and preventing dementia.

## Materials and Methods

All materials and methods have been published and more detailed information can be found in Sonntag et al. (2017) and Ryu et al. (2021).

### Subject Populations and Cell Lines

Subjects and cell lines are listed in Supplementary Table 1. Subjects were recruited at the McLean Hospital Memory Diagnostic Clinic and diagnosed by a geriatric psychiatrist using the Diagnostic and Statistical Manual of Mental Disorders (DSM-IV) criteria and the Montreal Cognitive Assessment (MOCA) score or the Hachinsky score (HIS) as indicated.

Fibroblasts were cultured as described (McPhie et al., 2018) and passaged three times before assaying. All efforts were made to keep equal passages between cell lines. iPSC lines were generated, cultured, and differentiated to NPCs and astrocytes as published in Ryu et al. (2021). All iPSC lines were propagated to passage 20 before differentiation. For assaying, NPCs were used at passages 8–14 and astrocytes at day *in vitro* (DIV) 30–40. Passage numbers and DIV did not impact data output.

### Data Collection

Data collection included previously published (Sonntag et al., 2017; Ryu et al., 2021) and newly generated results from existing or new cell lines (see Supplementary Table 1). Technical and experimental replicates are as stated below. For studies on iPSC-derived NPCs and astrocytes, an additional *n* = 4 control cell lines were used. There were no differences in data outcome from fibroblast- or PBMC-derived iPSC-differentiated NPCs and astrocytes and the cells’ donors’ age had no apparent influence on iPSC growth, differentiation, and descendent cell phenotype and function.

### NAD/NADH Assays

Data were generated with the NAD/NADH-Glo^TM^ Assay Kit (Promega, Madison, WI, United States) using 2 × 10^5^ cells per well in 96 well culture dishes as described (Ryu et al., 2021). Data were normalized with protein measurement on lysed cells using the Pierce^TM^ BCA Protein Assay kit (Thermo Fisher, Waltham, MA, United States). In each assay, six technical repeats were performed, and cell lines were analyzed in 2–4 repeat experiments.

### Seahorse XFp Cell Mito Stress Test

A detailed description of the Seahorse assay can be found in Ryu et al. (2021). Briefly, 1 × 10^4^/well fibroblasts, 2 × 10^4^/well NPCs, and 1 × 10^4^/well astrocytes were plated in Seahorse culture dishes and grown in the respective media overnight. Assays were performed in XF assay medium in the presence of 10 mM glucose, 1 mM pyruvate, and 2 mM glutamine, at pH 7.4. For data normalization, cells were stained with CyQuant solution (Life Technologies–Thermo Fisher Scientific, Carlsbad, CA, United States) diluted in XF assay medium and incubated for 1 h at 37°C. Green fluorescence (excitation: 485/20, emission: 528/20) was measured using a Synergy HT BioTek plate reader (BioTek Instruments, Winooski, VT, United States). Data analysis was performed using the Seahorse XF^e^ Wave software. Each assay was performed in triplicate and cell lines were analyzed in 2–4 repeat experiments.

### Biolog Assay

Experiments were performed using the Biolog MitoPlate S-1 assays (Biolog, Hayward, CA, United States) using 6 × 10^5^/well NPCs and 4 × 10^4^/well astrocytes in 96 well culture dishes as described (Ryu et al., 2021). OD 590 was measured at various time points (0, 1, 2, 3, 4, 5, 6, 24, and 48 h) using a Synergy HT BioTek plate reader (BioTek). Measurements were normalized to and calculated as percent change from no substrate control. Each measurement was done in triplicate and cell lines were analyzed in two repeat experiments.

### Lactate Assay

Lactate measurements were done with the Lactate-Glo^TM^ Assay Kit (J5021, Promega, Madison, WI, United States) performed according to protocols provided by the manufacturer. NPCs were plated at a density of 6 × 10^4^/well and astrocytes at 3 × 10^4^/well in 96 well culture dishes and cell culture medium was collected after 24 h of incubation. For the extracellular measurement, lactate detection reagent was added to the medium sample in a 1:1 ratio. Luminescence was measured after 60 min incubation at room temperature using a Synergy HT BioTek plate reader (BioTek). To measure intracellular lactate concentrations, cold PBS was added to the cells. After washing, inactivation solution (0.6 N HCl, #H1758, Sigma) was added and cells were incubated for 5 min. Cells were then treated with neutralization solution (1 M Trizma^®^, #T1503, Sigma) to inactivate metabolism. Luminescence was measured after 1 h incubation with Lactate detection reagent and data were normalized with protein measurement on lysed cells using Pierce^TM^ BCA Protein Assay kit (Thermo Fisher, Waltham, MA, United States).

### Statistical Analysis

Data were plotted as mean ± standard error of the mean (SEM) using one-way analysis of variance (ANOVA) tests for independent measures utilizing the Social Science Statistics software^2^ or PRISM 8 for macOS Version 8.1.0. Differences of comparison were considered statistically significant when *p*-values were less than 0.05, while *p*-values between 0.05 and 0.1 were considered trend data.

## Data Availability Statement

The original contributions presented in the study are included in the article/Supplementary Material, further inquiries can be directed to the corresponding authors.

## Ethics Statement

The studies involving human participants were reviewed and approved by Mass General Brigham Institutional Review Board. The patients/participants provided their written informed consent to participate in this study.

## Author Contributions

K-CS and BC conceptualized and designed the studies, interpreted the data, and reviewed the literature. W-IR performed the experiments and reviewed the literature and the manuscript. K-CS and W-IR analyzed the data. K-CS wrote the manuscript. All authors read, contributed to, and approved the manuscript.

## Conflict of Interest

The authors declare that the research was conducted in the absence of any commercial or financial relationships that could be construed as a potential conflict of interest.

## Publisher’s Note

All claims expressed in this article are solely those of the authors and do not necessarily represent those of their affiliated organizations, or those of the publisher, the editors and the reviewers. Any product that may be evaluated in this article, or claim that may be made by its manufacturer, is not guaranteed or endorsed by the publisher.

## Footnotes

1
http://www.merriam-webster.com/dictionary/bioenergetics

2
http://www.socscistatistics.com/Default.aspx

## Supplementary Material

Supplementary Figure 1 NAD^+^ and NADH production in iPSC-derived NPCs and astrocytes from individual cell lines. Relative NAD^+^ and NADH levels measured as luminescence and RR of LOAD (*n* = 9, red bars) or Control (*n* = 9, black bars) NPCs, and LOAD (*n* = 9, orange bars) or Control (*n* = 9, gray bars) astrocytes. Data are means +/– SEM from 2 to 4 repeat experiments for each cell line. **p* < 0.1; ***p* < 0.05; ****p* < 0.01 using one-way ANOVA.

Supplementary Figure 2 Correlation of NAD^+^, NADH, and RR with age in fibroblasts. Pearson’s correlation coefficients of NAD**^+^** and NADH data from Figure 3D and RR with age for all Controls (YC + OC, dashed line, ages 21–82), YC (black, ages 21–37), OC (gray, ages 55–82), and LOAD (red, ages 56–81). *R* and *p* values are indicated.
