# Supplementary material for: Hypothesis and Theory: Characterizing Abnormalities of Energy Metabolism Using a Cellular Platform as a Personalized Medicine Approach for Alzheimer’s Disease
Source: Front Cell Dev Biol. 2021 Jul 30;9:697578. doi: 10.3389/fcell.2021.697578 (PMC8363296; doi:10.3389/fcell.2021.697578)
Supplement: Supplementary Figure 1 — NAD+ and NADH production in iPSC-derived NPCs and astrocytes from individual cell lines. Relative NAD+ and NADH levels measured as luminescence and RR of LOAD (n = 9, red bars) or Control (n = 9, black bars) NPCs, and LOAD (n = 9, orange bars) or Control (n = 9, gray bars) astrocytes. Data are means +/– SEM from 2 to 4 repeat experiments for each cell line. *p < 0.1; **p < 0.05; ***p < 0.01 using one-way ANOVA. [file Data_Sheet_1.pdf]

Hypothesis and Theory: Characterizing Abnormalities of Energy Metabolism Using a Cellular Platform as a Personalized Medicine Approach for Alzheimer's Disease

Woo-In Ryu[1,2,3], Bruce M. Cohen[1,3,4*], and Kai-C. Sonntag[1,2,3*]

[1]Department of Psychiatry, McLean Hospital, Harvard Medical School, Belmont, MA 02478, USA.

[2]Basic Neuroscience Division, McLean Hospital, Harvard Medical School, Belmont, MA 02478, USA

[3]Program for Neuropsychiatric Research, McLean Hospital, Harvard Medical School, Belmont, MA 02478, USA.

[4]Psychotic Disorders Division, McLean Hospital, Harvard Medical School, Belmont, MA 02478, USA.

*** Correspondence:**
Kai-C. Sonntag (ksonntag@mclean.harvard.edu) or Bruce M. Cohen (bcohen@mclean.harvard.edu)

Keywords: Alzheimer's Disease[1], Bioenergetics[2], Metabolism[3], Pluripotent Stem Cells[4], Cellular Platform[5], Disease Modeling[6], Personalized Medicine[7]

Supplementary Tables and Figures

Supplementary Table 1 | Sample list.

sample ID	sample ID iPSC	Age	Sex	Race	Diagnosis	APOE	Donor Cell iPSC	Karyotype	iPSC Characterization	iPSC Source	Reprogramming iPSC	Cell Types Analyzed	HIS*	MOCA*	MMSE*	Comorbidity	Reference
367		21	M	white	Ctrl	E3/3						Fibroblasts				No serious illness	Sonntag et al., 2017
368	C1	21	M	SE asian	Ctrl	E2/3	Dermal Fibroblast	normal, male	Yoshimizu 2015; McPhie 2018	NYSCF	RNA transfection	Fibroblasts, NPC, astrocytes				No serious illness	Ryu et al., 2021
294		23	M	white	Ctrl	E2/2						Fibroblasts				No serious illness	Sonntag et al., 2017
460		25	M	white	Ctrl	E4/4						Fibroblasts				No serious illness	Sonntag et al., 2017
458		26	M	white	Ctrl	E2/3						Fibroblasts				No serious illness	Sonntag et al., 2017
359		31	M	black	Ctrl	E3/4						Fibroblasts				No serious illness	Sonntag et al., 2017
298		33	M	black	Ctrl	E2/3						Fibroblasts				No serious illness	Sonntag et al., 2017
366		35	M	white	Ctrl	E3/3						Fibroblasts				No serious illness	Sonntag et al., 2017
15	C2	37	M	white	Ctrl	E3/3	Dermal Fibroblast	normal, male	Yoshimizu 2015; McPhie 2018	Tsai Lab	RNA transfection	Fibroblasts, NPC, astrocytes				No serious illness	Ryu et al., 2021
152		40	M	black	Ctrl	E3/3						Fibroblasts				No serious illness	Sonntag et al., 2017
190		42	M	white	Ctrl	E3/3						Fibroblasts				No serious illness	Sonntag et al., 2017
465		44	M	white	Ctrl	E3/3						Fibroblasts				No serious illness	
226		45	M	white	Ctrl	E3/3						Fibroblasts				No serious illness	Sonntag et al., 2017
135		49	M	white	Ctrl	E3/3						Fibroblasts				No serious illness	Sonntag et al., 2017
365		52	M	white	Ctrl	E3/3						Fibroblasts				No serious illness	Sonntag et al., 2017
316		54	M	white	Ctrl	E3/3						Fibroblasts				No serious illness	Sonntag et al., 2017
14		55	M	white	Ctrl	E3/3						Fibroblasts				No serious illness	Sonntag et al., 2017
342	C3	57	M	white	Ctrl	E3/4	Dermal Fibroblast	normal, male	Ryu et al., 2021	UNC	Sendai virus	Fibroblasts, NPC, astrocytes				No serious illness	Sonntag et al., 2017, Ryu et al., 2021
44	C6	59	M	white	Ctrl	E3/3	Dermal Fibroblast	normal, male		UNC	Sendai virus	Fibroblasts, NPC, astrocytes				No serious illness, hypertension, cholesterol-reducing meds	
498	C4	60	F	white	Ctrl	E3/3	Dermal Fibroblast	normal, female	Ryu et al., 2021	UNC	Sendai virus	Fibroblasts, NPC, astrocytes				No serious illness	Ryu et al., 2021
491	C5	61	M	white	Ctrl	E3/4	Dermal Fibroblast	normal, male	Ryu et al., 2021	UNC	Sendai virus	Fibroblasts, NPC, astrocytes				No serious illness	Sonntag et al., 2017, Ryu et al., 2021
497		61	F	white	Ctrl	E3/3						Fibroblasts				No serious illness	
127		62	M	white	Ctrl	E3/4						Fibroblasts				Diabetes II, high BP, brain tumor	Sonntag et al., 2017
357		65	M	white	Ctrl	E3/3						Fibroblasts				No serious illness	Sonntag et al., 2017
47		65	F	white	Ctrl	E3/3						Fibroblasts				No serious illness	
569		66	M	white	Ctrl	E3/3						Fibroblasts				No serious illness, rheumatoid arthritis on ibuprofen	
205		67	M	white	Ctrl	E3/4						Fibroblasts				No serious illness, rheumatoid arthritis on ibuprofen	Sonntag et al., 2017
140	C7	72	M	white	Ctrl	E3/3	Dermal Fibroblast	normal, male		UNC	Sendai virus	Fibroblasts, NPC, astrocytes				No serious illness	
405	C8	75	F	white	Ctrl	E3/3	Dermal Fibroblast	normal, female		UNC	Sendai virus	Fibroblasts, NPC, astrocytes				No serious illness, hypertension	Sonntag et al., 2017
169	C9	82	M	white	Ctrl	E3/3	Dermal Fibroblast	normal, male		UNC	Sendai virus	Fibroblasts, NPC, astrocytes				No serious illness	
563		88	M	white	Ctrl	E3/3						Fibroblasts				No serious illness	
330	AD1	56	M	white	AD	E3/3	Dermal Fibroblast	normal, male	Ryu et al., 2021	Tsai Lab	Sendai virus	Fibroblasts, NPC, astrocytes	0	24		No serious illness	Sonntag et al., 2017, Ryu et al., 2021
374		59	F	white	AD	E3/4						Fibroblasts	1	14		No serious illness	Sonntag et al., 2017
580		63	M	white	AD	E3/3						Fibroblasts	NA	NA	6	Diabetes II	
572		64	F	white	AD	E3/4						Fibroblasts	2	6		No serious illness	
231	AD2	65	M	white	AD	E3/4	Dermal Fibroblast	normal, male	Ryu et al., 2021	Tsai Lab	Sendai virus	Fibroblasts, NPC, astrocytes	NA	NA		Glaucoma, hypertension, CAD, cerebrovascular accident	Sonntag et al., 2017, Ryu et al., 2021
311		70	M	white	AD	E4/4						Fibroblasts	NA	NA		No serious illness	Sonntag et al., 2017
402		70	F	white	AD	E3/4						Fibroblasts	2	18		Not recorded	Sonntag et al., 2017
399	AD3	71	F	white	AD	E4/4	Dermal Fibroblast	normal, female	Ryu et al., 2021	Tsai Lab	Sendai virus	Fibroblasts, NPC, astrocytes	2	10		No serious illness	Sonntag et al., 2017, Ryu et al., 2021
BC2	AD7	71	M	white	AD	E3/4	PBMC	normal, male	Ryu et al., 2021	UNC	Sendai virus	NPC, astrocytes	0	NA	23	No serious illness	Ryu et al., 2021
566		74	F	white	AD	E3/4						Fibroblasts	3	22		Hemochromatosis	
369	AD4	76	M	white	AD	E3/4	Dermal Fibroblast	normal, male	Ryu et al., 2021	Tsai Lab	Sendai virus	Fibroblasts, NPC, astrocytes	1	13		Hemochromatosis	Sonntag et al., 2017, Ryu et al., 2021
281	AD5	79	M	white	AD	E4/4	Dermal Fibroblast	normal, male	Ryu et al., 2021	Tsai Lab	Sendai virus	Fibroblasts, NPC, astrocytes	4	18		Diabetes II, hyperlipidemia, hypertension, CAD	Sonntag et al., 2017, Ryu et al., 2021
404	AD6	81	M	white	AD	E3/4	Dermal Fibroblast	normal, male	Ryu et al., 2021	Tsai Lab	Sendai virus	Fibroblasts, NPC, astrocytes	1	5		No serious illness	Sonntag et al., 2017, Ryu et al., 2021
BC3	AD8	78	F	white	AD	E3/4	PBMC	normal, female	Ryu et al., 2021	UNC	Sendai virus	NPC, astrocytes	2	NA	27	Diabetes II, hypertension, hyperlipidemia	Ryu et al., 2021
332		82	F	white	AD	E3/3						Fibroblasts	1	14		No serious illness	Sonntag et al., 2017
BC4	AD9	>89	F	white	AD	E3/4	PBMC	normal, female	Ryu et al., 2021	UNC	Sendai virus	NPC, astrocytes	MRI	3		No serious illness	Ryu et al., 2021

*HIS: Hachinsky Score
*MOCA: Montreal Cognitive Assessment Test
*MMSE: Mini-Mental State Exam

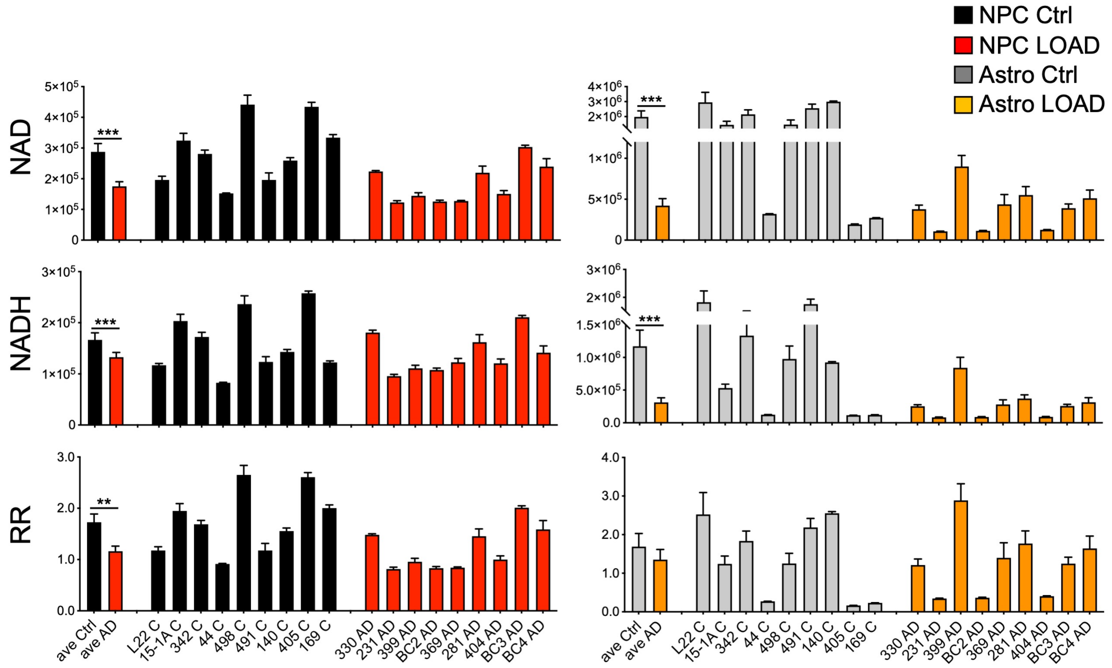

Supplementary Figure S1 | NAD+ and NADH production in iPSC-derived NPCs and astrocytes from individual cell lines. Relative NAD+ and NADH levels measured as luminescence and RR of LOAD (*n* = 9, red bars) or Control (*n* = 9, black bars) NPCs, and LOAD (*n* = 9, orange bars) or Control (*n* = 9, grey bars) astrocytes. Data are means +/- SEM from 2-4 repeat experiments for each cell line. **p* < 0.1; ***p* < 0.05; ****p* < 0.01 using one-way ANOVA.

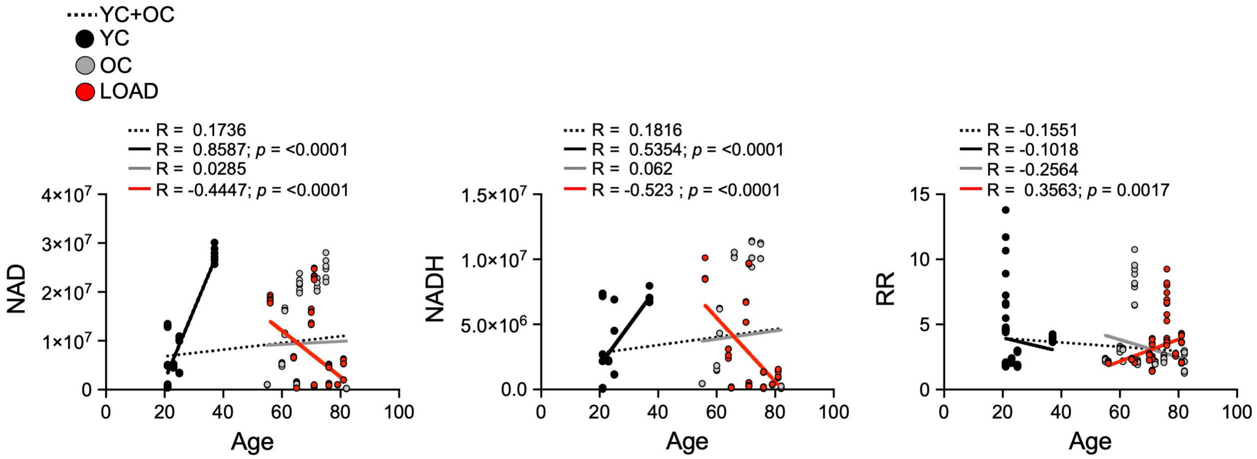

Supplementary Figure S2 | Correlation of NAD^+, NADH, and RR with age in fibroblasts. Pearson's correlation coefficients of NAD^+ and NADH data from Fig. 3D and RR with age for all Controls (YC+OC, dashed line, ages 21 – 82), YC (black, ages 21 – 37), OC (grey, ages 55 – 82), and LOAD (red, ages 56 – 81). R and *p* values are indicated.
